# Visualization of kidney fibrosis in diabetic nephropathy by long diffusion tensor imaging MRI with spin-echo sequence

**DOI:** 10.1038/s41598-017-06111-4

**Published:** 2017-07-18

**Authors:** Jun-Ya Kaimori, Yoshitaka Isaka, Masaki Hatanaka, Satoko Yamamoto, Naotsugu Ichimaru, Akihiko Fujikawa, Hiroshi Shibata, Akira Fujimori, Sosuke Miyoshi, Takashi Yokawa, Kagayaki Kuroda, Toshiki Moriyama, Hiromi Rakugi, Shiro Takahara

**Affiliations:** 1 Department of Advanced Technology for Transplantation, Osaka University Graduate School of Medicine, 2-2 Yamadaoka, Suita, Osaka 565-0781 Japan; 2 Department of Geriatric Medicine and Nephrology, Osaka University Graduate School of Medicine, 2-2 Yamadaoka, Suita, Osaka 565-0871 Japan; 3 Drug Discovery Research, Astellas Pharma Inc., 21 Miyukigaoka, Tsukuba, Ibaraki 305-8585 Japan; 4 BioView Inc., 2-16-16 Iwamoto-cho, Chiyoda-ku, Tokyo 101-0032 Japan; 5 Department of Human and Information Science, Tokai University School of Information Science and Technology, 4-1-1 Kitakaname, Hiratsuka, Kanagawa 259-1292 Japan; 6 Osaka University Health Care Center, 2-1 Yamadaoka, Suita, Osaka 565-0871 Japan

## Abstract

Renal fibrosis (RF) is an indicator for progression of chronic kidney disease (CKD). Although diabetic nephropathy (DN) is the leading cause of CKD and end-stage renal disease in Western populations, the ability of MRI to evaluate RF in DN patients has not been determined. As a first step to identify possible MRI methods for RF evaluation, we examined the use of diffusion tensor imaging (DTI) MRI to evaluate RF in a rat model of DN (SHR/NDmcr-cp(cp/cp): SHR/ND). The signal-to-noise ratio in DTI MRI was enhanced using a spin-echo sequence, and a special kidney attachment was developed for long-term stabilization. The changes in renal temperature and blood flow during measurement were minimal, suggesting the feasibility of this method. At 38 weeks of age, RF had aggressively accumulated in the outer stripe (OS) of the outer medulla. FA maps showed that this method was successful in visualizing and evaluating fibrosis in the OS of the SHR/ND rat kidney (r = 0.7697, P = 0.0126). Interestingly, in the FA color maps, the directions of water molecule diffusion in RF were random, but distinct from conventional water diffusion in brain neuron fibers. These findings indicate that DTI MRI may be able to evaluate RF in CKD by DN.

## Introduction

Chronic kidney disease (CKD) is a worldwide health problem and a risk factor for cardiovascular events. Diabetic nephropathy (DN) is the leading cause of CKD and end-stage renal disease (ESRD) in Japanese and Western populations^1^. Renal fibrosis (RF) has been shown to be correlated with loss of renal function and poor renal prognosis^2, 3^. At present, invasive biopsy is the only method for accurate evaluation of RF. Despite the associations of DN with CKD and ESRD^4, 5^, a renal biopsy in diabetes patients is only indicated for those suspected of having nephropathies other than DN^6^.

While MRI was previously used to assess structural changes in the kidneys, it is currently used to evaluate physiological aspects of the kidneys, including renal tissue oxygenation and perfusion^7^. Diffusion-weighted MRI (DWI), in which water molecules are mobilized and assessed by MRI, has been successfully used in the kidneys, because the main kidney functions are all related to water movement, such as glomerular filtration, secretion, and passive and active reabsorption by tubules^8^. DWI has been used to evaluate tissue injury, including extracellular and intracellular edema, in kidney grafts from cardiac-death donors^9^. DWI has also been used to assess RF in mouse models of kidney fibrosis caused by unilateral urethral obstruction (UUO) and in non-diabetic CKD patients, revealing significant negative correlations between fibrosis markers and apparent diffusion coefficients (ADCs)^10, 11^. However, in a rat model of RF caused by UUO, the ADC did not reflect the extent of RF^12^, suggesting that the ADC alone cannot be used for evaluation of RF in all kidney diseases. Diffusion tensor imaging (DTI), a DWI technique, is used to visualize the brain fiber structure by enhanced signals in a fractional anisotropy (FA) map^13, 14^. This is because DTI can differentiate water molecule diffusivities both along and against neuronal fibers. Consequently, it is easy to speculate that this MRI technique may also be able to detect RF, by analogy to its detection of neuronal fibers in the brain. To date, DTI has been trialed for the evaluation of kidney tissue damage in CKD patients^15^, including glomerulonephritis^16^, diabetic nephropathy^17^, and allograft kidney injury^18^. However, in all of these studies, the kidney tissue FA values were decreased, rather than being enhanced as observed in the brain, and the correlation between signal intensity reduction and RF was obscure. Furthermore, the FA value in the inner medulla (IM) of rats with DN plus unilateral nephrectomy was found to be negatively correlated with interstitial fibrosis^19^. This body of evidence suggests that it is very difficult to visualize and evaluate RF using DTI in a similar manner to neuronal fibers, particularly in DN tissue where intracellular or interstitial edema affects the manner of water molecule diffusion. Previously, we developed a long DTI MRI method with spin-echo sequence to evaluate RF in UUO model rats^20^. Here we report successful visualization of RF in diabetic rats with enhanced fibrosis (SHR/NDmcr-cp(cp/cp): SHR/ND) using our DTI MRI method with spin-echo sequence and long duration measurement enabled by a special kidney attachment.

## Results

### Characterization of SHR/ND rats

The SHR/ND rat is a diabetic rat model with concentrated and enhanced RF^21^. As a pilot study, we examined the disease phenotypes of SHR/ND rats. SHR/ND rats showed extreme obesity and hypertension, with the kidney tissue of older rats showing massive fibrotic changes concentrated in the outer stripe (OS) of the outer medulla (Supplementary Fig. S1a,b). Oral administration of the antihypertensive drug telmisartan at 5 mg/kg/day from 8–38 weeks of age to improve the hypertension and RF^22^ dramatically reduced these fibrotic changes (Supplementary Fig. S1a,b).

### Confirmation of feasibility of long DTI MRI measurement with a special attachment

**Figure 1:**
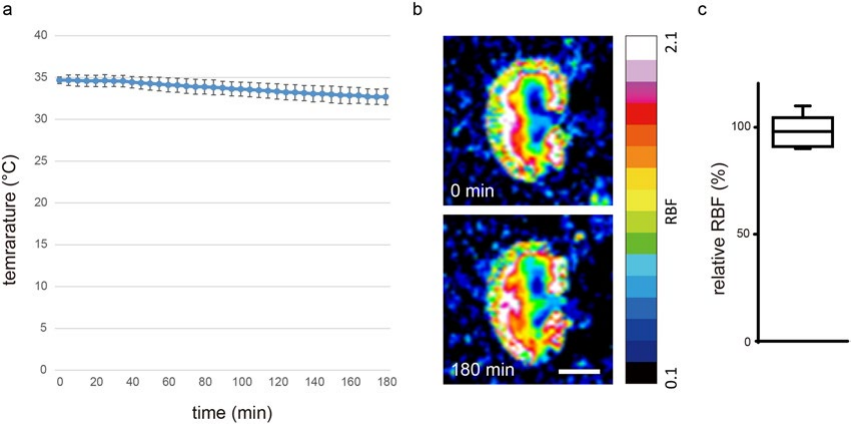
Confirmation of feasibility of long DTI MRI measurement with a special attachment. (**a**) Temperature changes in kidneys folded by the attachment during 180 min. (**b**) MR images of renal blood flow at 0 min (upper) and 180 min (lower) after setting of the attachment. (**c**) Quantification of relative renal blood flow based on the renal blood flow at 0 min. The data are shown as mean ± SD.

We speculated that our previously developed long DTI MRI method with spin-echo sequence^20^ would detect the concentrated RF in 38-week-old SHR/ND rats, based on the results of our pilot study. However, the rats were so obese that it was very difficult to hold and fix the kidney from outside of the body using the previous attachment. Therefore, we developed a semi-invasive method to fold and retain the kidney in a humid environment in these obese rats (Supplementary Fig. S2a). To confirm the feasibility of this DTI MRI system, we examined renal perfusion and temperature in kidneys held by the attachment for 3 h. During the 3 h, the renal temperature gradually reduced from 34.68 ± 0.40 °C to 32.70 ± 0.97 °C (Fig. 1a), but the change was not significant (n = 5; Wilcoxon matched test, p = 0.062). The relative renal blood flow at 3 h based on 0 h was 97.8 ± 7.8% (n = 5) measured using an Super Paramagnetic Iron Oxide (SPIO) (Fig. 1b,c). The relative renal blood flow data between 0 h and 3 h also showed no significant difference (one-sample *t*-test, p = 0.56).

### DTI MRI evaluation of RF in DN model rats at 8 weeks of age

The FA value of the IM on DTI MRI was found to be the only parameter associated with total RF, with the FA values in rats with DN plus unilateral nephrectomy being decreased in the cortex (CO) and OS of the medulla^19^. These findings suggested that MRI would be sufficiently sensitive to detect RF, as shown by the total decrease in the FA value in rats with DN plus unilateral nephrectomy.

**Figure 2:**
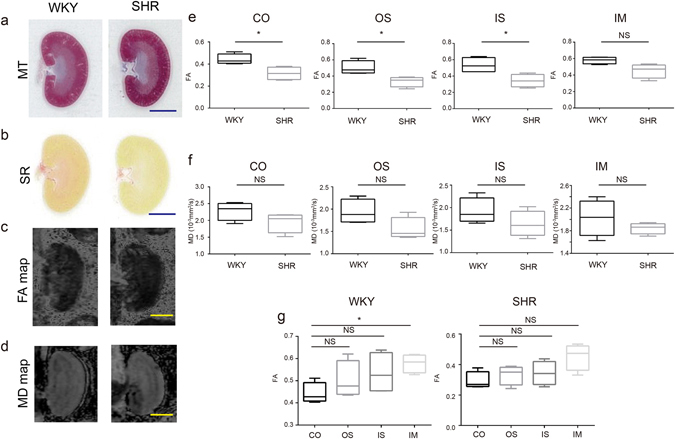
Analyses of FA and MD values in the kidneys of 8-week-old WKY and SHR/ND rats. (**a**–**d**) Masson trichrome (MT) (**a**), Sirius Red (SR) (**b**), FA map (**c**), and MD map (**d**) of WKY (left panel) and SHR/ND (right panel) rat kidneys. Scale bars, 5 mm. (**e**,**f**) Quantification of FA (**e**) and MD (**f**) values in the CO, OS of outer medulla, IS of outer medulla, and IM of WKY and SHR/ND rat kidneys. *p < 0.05, WKY vs SHR. (**g**) FA values in the same portions of WKY (left panel) and SHR/ND (right panel) rat kidneys. The data are shown as mean ± SD.

At 8 weeks of age, SHR/ND rats had a higher body weight, more profound hypertension, lower renal function, more elevated serum glucose, and more urine protein than control WKY/ism (WKY) rats (Table 1). However, the degree of RF was similar in the kidney tissues of 8-week-old SHR/ND and WKY rats (Figs 2a,b and S3a–d). Examination of these kidneys by DTI MRI showed that the calculated FA values in the CO, OS, and inner stripe (IS) of the outer medulla were lower in SHR/ND rats compared with WKY rats, while the FA values in the IM did not differ significantly.

**Table 1:** Physiological data of WKY/ism and SHR/ND rats at 8 weeks of age.

	WKY/ism (n = 7)	SHR/ND (n = 7)	
Body weight (g)	264.4 ± 7.1	343.5 ± 7.3	P = 0.0021
SBP (mmHg)	137.0 ± 12.5	152.1 ± 23.5	P = 0.2496
DBP (mmHg)	94.0 ± 17.3	110.6 ± 17.0	P = 0.1788
HR (/min)	395 ± 31	388 ± 43	P = 0.7489
Urine Volume (ml/day)	18.0 ± 4.8	27.3 ± 4.6	P = 0.0072
Serum Cr (mg/dl)	0.202 ± 0.01	0.166 ± 0.01	P = 0.0019
Ccr (ml/min/kg)	10.7 ± 1.5	8.3 ± 0.7	P = 0.0023
Serum BUN	13.9 ± 1.8	28.7 ± 1.7	P = 0.0021
Serum Gluc	145.9 ± 5.4	216.0 ± 51.3	P = 0.0213
Urine protein (mg/day)	128.1 ± 47.6	184.9 ± 36.3	P = 0.0262

### DTI MR evaluation of RF in DN model rats at 38 weeks of age

**Figure 3:**
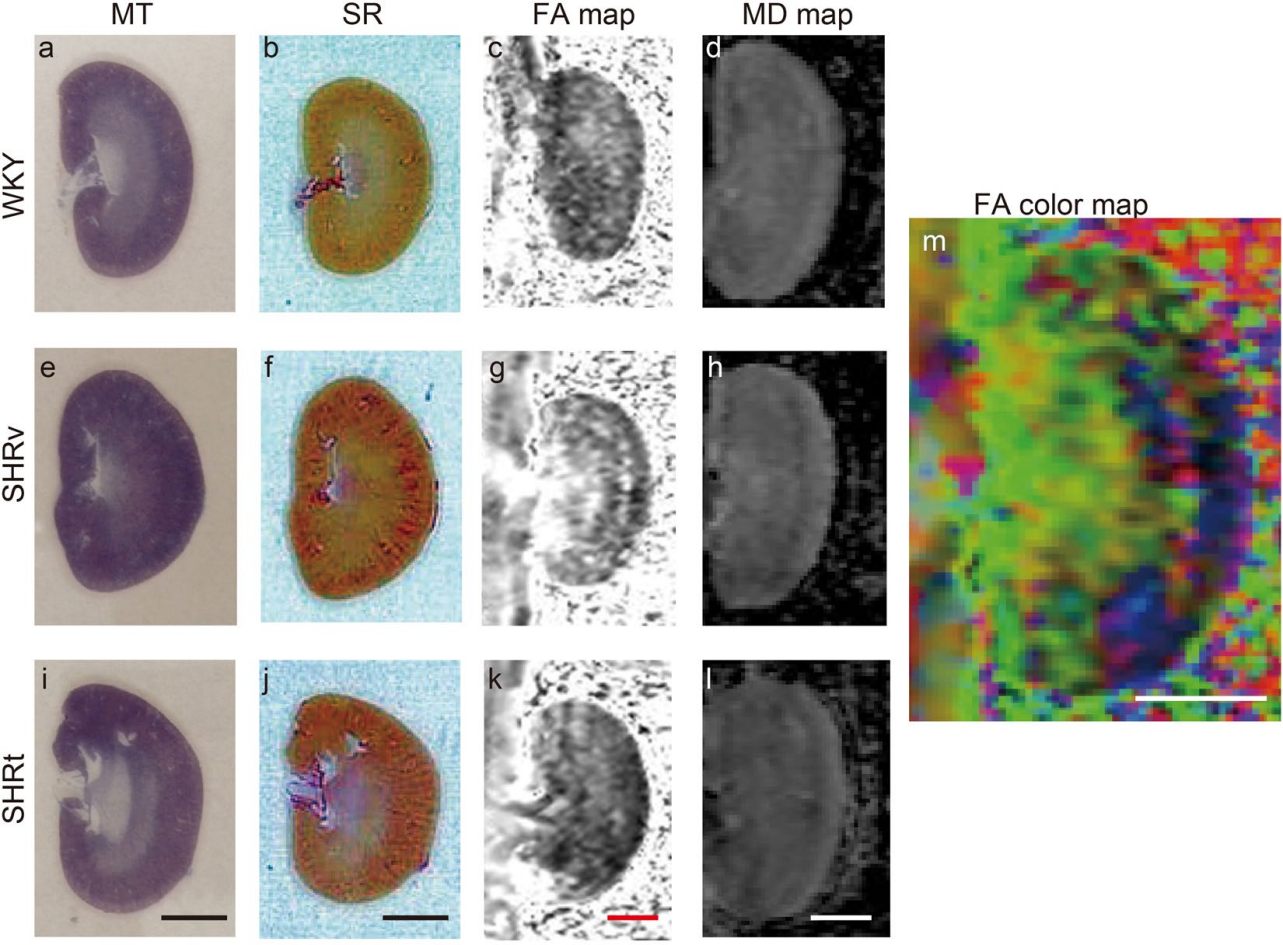
Visualization of RF in FA maps. (**a**–**l**) Images of Masson Trichrome (MT) (**a**,**e**,**i**), Sirius Red (SR) (**b**,**f**,**j**), FA map (**c**,**g**,**k**), and MD map (**d**,**h**,**l**) in kidneys from WKY rats, SHR/ND rats treated with vehicle (SHRv), and SHR/ND rats treated with telmisartan (SHRt). (**m**) FA color map of an SHRv kidney. Scale bars, 5 mm.

**Figure 4:**
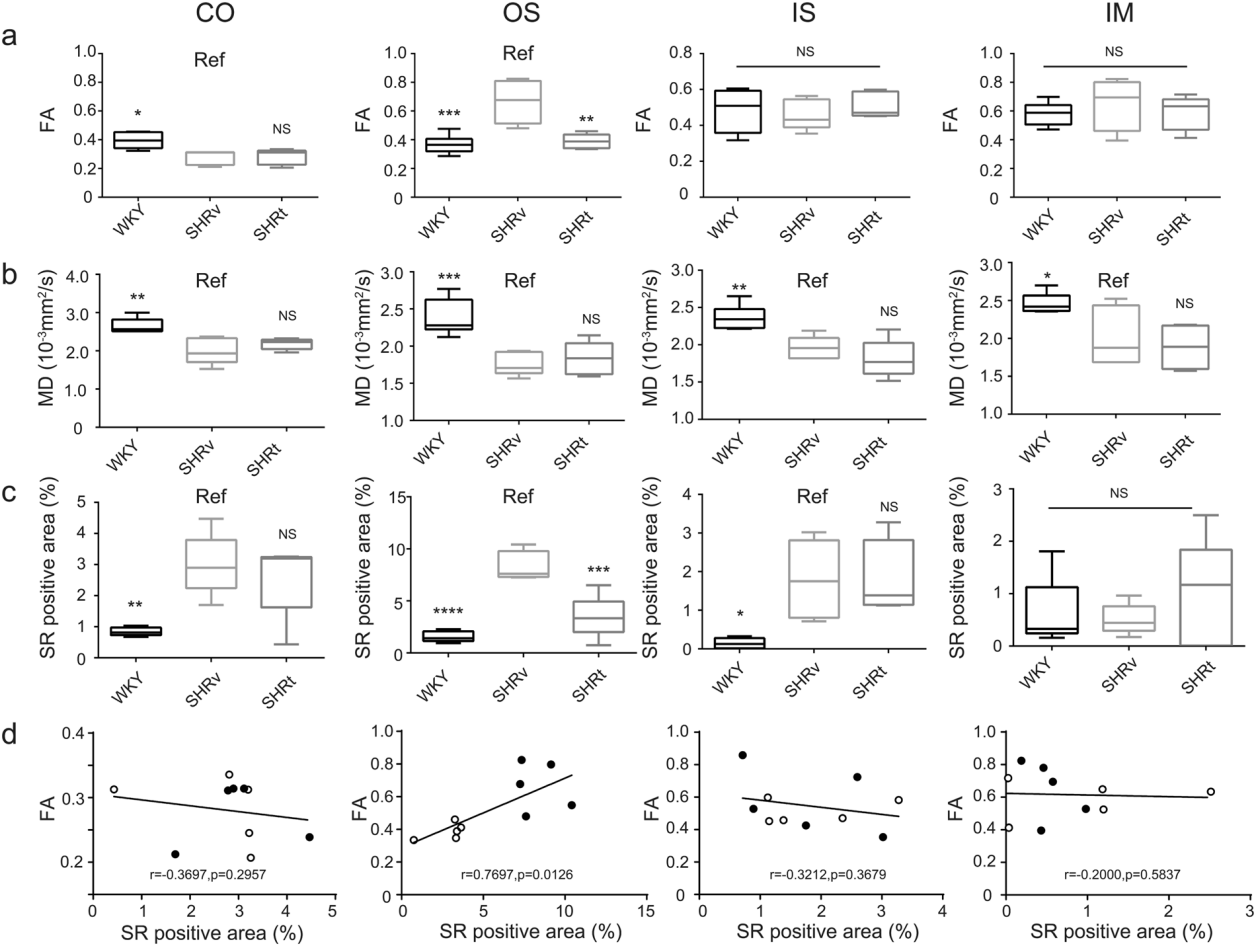
Quantification of FA, MD values, and RF. (**a**–**c**) Quantification of FA (**a**), MD values (**b**), and RF measured by Sirius Red (SR) staining (**c**) in the CO, OS of outer medulla, IS of outer medulla, and IM of kidneys from WKY rats, SHR/ND rats treated with vehicle (SHRv), and SHR/ND rats treated with telmisartan (SHRt). SHR rats were treated with vehicle vs telmisartan. The data are shown as mean ± SD. *p < 0.05, **p < 0.01, ***p < 0.001, SHRv vs WKY or SHRt. (**d**) Correlations of FA values with fibrotic areas measured by SR staining based on the data for SHRv and SHRt rat kidneys only. Open circles and closed circles indicate the data from SHRt and SHRv rats respectively.

SHR/ND rats showed more profound metabolic syndrome phenotypes at 38 weeks of age than at 8 weeks of age (Table 2), with the kidney tissue of older rats showing massive fibrotic changes concentrated in the OS (Figs 3e,f and S4b). Oral administration of the antihypertensive drug telmisartan at 5 mg/kg/day from 8–38 weeks of age to improve RF^22^ dramatically reduced these fibrotic changes (Figs 3i,j and 4c and S4d–j). Consistent with the concentrated fibrotic changes in untreated SHR/ND rats, the FA signals were sufficiently high to be visible in the OS of the kidneys in the same animals (Fig. 3e–g), but were not observed in WKY rats or SHR/ND rats treated with telmisartan (Fig. 3c,k). Furthermore, the directions of FA in fibrotic areas of the OS in untreated SHR/ND rats appeared to be random (Fig. 3m), being distinct from the FA color map images of nerve fibers in the brain^23^. The FA values of the OS were higher in SHR/ND rats than in other rats, and were higher in the OS than in other portions of the same kidneys (Figs 4a and S4k–m). The FA values of the CO were reduced in SHR/ND rats with or without telmisartan, consistent with the results at 8 weeks of age and with previous findings^19, 24^. The mean diffusivity (MD) looked similar in all groups of rats (Fig. 3d,h,i), but the quantified MD values were lower in the CO, OS, and IS of SHR/ND rats with or without telmisartan than in WKY rats (Fig. 4b). Based on the differences in FA values between the kidneys of WKY and SHR/ND rats, we analyzed the data from SHR/ND rats with and without telmisartan treatment only. We observed a significant correlation between the fibrotic area measured by Sirius Red staining and the FA value only in the OS (r = 0.7697, P = 0.0126) (Fig. 4d). Even if we included the data of WKY rats in addition to Fig. 4d, we still observed a significant correlation in OS (r = 0.7812, p = 0.0004) with the tendencies of other compartments not changed.

**Table 2:** Physiological data of WKY/ism and SHR/ND rats at 38 weeks of age.

	WKY/ism (n = 8)	SHR/ND vehicle (n = 5)	SHR/ND telmisartan (n = 5)
Body weight (g)	467.5 ± 18.1	801.7 ± 18.1^a,^***	770.3 ± 27.2^a,^***
SBP (mmHg)	131.1 ± 10.3	166.8 ± 22.3^a,^**	121.4 ± 5.8^b,^***
DBP (mmHg)	101.3 ± 6.1	132.6 ± 12.5^a,^***	98.4 ± 14.1^b,^***
HR (/min)	342 ± 24	395 ± 77	446 ± 14^a,^**
Urine Volume (ml/day)	20.3 ± 6.4	22.4 ± 1.8	36.0 ± 6.9^a,^***^,b,^**
Serum Cr (mg/dl)	0.500 ± 0.137	0.300 ± 0.043	0.300 ± 0.146
Ccr (ml/min/kg)	5.3 ± 1.1	3.2 ± 0.5^a,^**	3.7 ± 1.2^a^
Serum BUN	8.7 ± 3.5	24.8 ± 2.2^a,^*	26.5 ± 3.8^a^
Serum Gluc	141.6 ± 9.4	268.0 ± 46.5^a,^***	259.0 ± 28.3^a^
Urine protein (mg/day)	111.3 ± 24.7	1587.5 ± 140.1^a,^***	380 ± 46.7^a,^***^,b,^***

^a^WKY vs, ^b^SHR/ND (vehicle) vs, *p < 0.05, **p < 0.01, ***p < 0.001.

## Discussion

This study identified an MRI method that can visualize RF, even in the presence of DN. ADC values calculated from diffusion MRI data were reported to accurately indicate RF in patients with CKD^11^ and in a mouse UUO model^10^. As the kidneys handle considerable volumes of water to maintain body fluid volumes, their ADC values are likely to be heavily affected by circulation and urine currents^12^. Indeed, the ADC values in human kidneys were reported to be highly variable^25^. FA values are calculated from DTI MRI data, and performed in six different directions. This study used a spin-echo sequence, rather than the echo-planar imaging method conventionally used in DTI MRI, to enhance the signal-to-noise ratio^26^. Therefore, the MRI required 3 h for completion. To keep the kidney still during this time period without tissue compression, we devised a special procedure and device to isolate and fix the kidney, even in an extremely obese rat. To confirm the feasibility of this DTI MRI system, we examined the kidney temperature and blood flow during 3 h under the same settings used for the DTI MRI. We found that the changes in kidney temperature and blood flow during 3 h were not significant. In addition, we also selected an approximately-unbiased and automated method for measuring RF (Supplementary Fig. S6) for reliability for data sampling.

Although a previous study attempted to evaluate histopathological changes by DTI MRI in a rat model of DN induced by streptozotocin and uninephrectomy, the FA values in the CO, OS, and IM of DN rats following uninephrectomy were not linearly correlated with tissue fibrosis^19^. We suspected that the increase in FA was undetectable because these rats experienced only modest fibrotic changes. Moreover, at the low b-value (300 s/mm^2^) used, MR images are easily influenced by perfusion and flow effects. The present study utilized a rat DN model with more aggressive RF and a higher b-value (601 s/mm^2^), resulting in the detection of increased FA in the fibrotic kidney tissues and visualization of RF in rats with DN. The striking difference between the FA values in the CO and OS of the kidneys in SHR/ND rats may make the FA signal increases more visible. Similarly, use of a higher b-value (1000 s/mm^2^) in a previous study resulted in FA being significantly higher in the presence of massive liver fibrosis compared with modest liver fibrosis (4 vs. 2 weeks after CCl_4_ insult)^27^. Although the mechanisms underlying the decreased diffusion anisotropy in DN tissues remain to be determined^17, 19^, tissue edema may be a possible cause. Interestingly, the brain tissue in fulminant hepatic failure exhibits massive edema, which was reported to be a cause of the reduction in its FA value^28^. Our DTI MRI method could successfully detect enhanced signals in the aggressive RF area, suggesting that fibrotic tissue changes may increase FA values even in DN with edema. In contrast, the MD values in this study differed from those in a previous report^19^. Although the reasons for the discrepancies were not determined, they may arise through differences in the model animals or b-values. In the future, it will be interesting to measure multiple ADC components^29^ in DN rats with RF.

In rat experiments, our DTI MRI method requires a very long time and is partially invasive owing to oscillation of the kidney and its surrounding fat. For studies in humans, the influence of oscillation by breathing is preventable by respiratory/pulse triggering measurement. However, we need to devise a more sensitive sequence to shorten the duration of measurement.

## Conclusions

DTI MRI may be able to visualize RF in DN patients, if we can devise a suitable MRI sequence that can be applied in real clinical scenarios and has good sensitivity in the future.

## Methods

### Animals

The present experiments were performed at three sites: Institute of Experimental Animal Sciences of Osaka University Medical School (Osaka, Japan), Business Support Center for Biomedical Research Activities (Hyogo, Japan), and Minami-Yamashiro Laboratory of Oriental Bioservice Inc. (Kyoto, Japan). Male SD rats weighing 250–300 g were purchased from Oriental Bioservice Inc., and WKY and SHR/ND rats were provided by the Disease Model Cooperative Research Association (Kyoto, Japan). The rats in all three facilities were kept in strictly controlled environments under fixed temperature, humidity, and 12-h/12-h light/dark cycle, and were provided with standard laboratory chow and water *ad libitum*. All experiments were performed according to established guidelines for animal welfare and were approved by the Animal Ethics Committee of Osaka University (Approval Number: DOI 22-057-0).

### Experimental disease model and design

Seven-week-old WKY and SHR/ND rats were allowed to acclimate for 7 days. Seven WKY rats and five SHR/ND rats were treated p.o. with vehicle (0.5% methyl cellulose; Wako Pure Chemical Industries Ltd., Osaka, Japan), and five SHR/ND rats were treated with 5 mg/kg/day telmisartan (Tokyo Chemical Industry Co. Ltd., Tokyo, Japan) suspended in 0.5% methyl cellulose from 8–38 weeks of age. Body weight, blood pressure, heart rate, urine volume, and urine protein excretion were measured weekly. After each examination, rats were euthanized and kidney samples were harvested for measurements of fibrotic markers. As a pilot study, we conducted almost the same experimental procedure without MRI measurements using WKY rats (n = 9), SHR/ND rats treated with vehicle (n = 9), and SHR/ND rats treated with telmisartan (n = 9).

### Attachment for MRI of the kidneys

The devised attachment (Supplementary Fig. S2a) was used in hugely obese SHR/ND rats, in which the kidneys were surrounded by large amounts of visceral fat. The skin around the left kidney was cut and the kidney was carefully isolated from the surrounding fat tissue, taking care not to induce additional tissue injury or bleeding.

### MRI of the kidneys of DN rats

Before MRI measurements, the rats remained drug-free for about 4 days to wash out antihypertensive drug or vehicle. MR images were obtained using a Unity INOVA MR spectrometer (Varian Associates Inc., Palo Alto, CA, USA) with a JASTEC Horizontal Magnet 4.7 T (JMTB-4.7/310/SS; Japan Superconductor Technology Inc., Hyogo, Japan). In SHR/ND rats, a rabbit volume coil (Takashima Seisakusho Co. Ltd., Tokyo, Japan) was used for transmission, along with an in-house-made surface coil (Supplementary Fig. S2b). All measurements were performed in a 20 °C room. The rats were sedated by inhalation of mixed gas (O_2_ 0.35 L/min, N_2_O 0.15 L/min, 1.5% isoflurane; Mylan Inc., Canonsburg, PA, USA). All images were acquired without respiratory or pulse triggering. To compare different imaging sequences, images were acquired in the same geometry for the four portions, namely the CO, OS of outer medulla, IS of outer medulla, and IM.

### Measurement of relative renal blood flow by MRI using a SPIO

Sagittal 2D T2*-weighted images were acquired with the following parameters: repetition time (TR) = 12 ms; echo time (TE) = 6.0 ms; flip angle = 30°; field of view (FOV) = 120 mm × 60 mm; matrix = 256 × 64 to 256 × 128 by zero-fill; slice thickness = 2 mm. After about 20 baseline images, the SPIO (Ferucarbotran; Resovist, Schering, Berlin, Germany) was injected as a bolus at the dose of 100 μmol Fe/kg and the perfusion images were monitored for 2 min 24 s (180 phases of 0.8 s). For relative renal blood flow measurements, 200 MRI data sets were continuously obtained for 160 s (0.8 s/image). Renal blood volume, mean transit time, and renal blood flow were calculated using the 20 data sets before SPIO administration as baseline and the data sets between the abrupt signal decrease observed at the kidney artery and the minimum point of the signal intensity induced by the SPIO.

### Measurement of kidney temperature in the attachment

Kidney temperature was measured every 1 s for 180 min using a fiberoptic thermometer (FL-2000; Anritsu Meter Co. Ltd., Tokyo, Japan). The fiber probe was a Model FS100 (Anritsu Meter Co. Ltd.) calibrated at 37.0 °C. We set time 0 as 3 min after the setting was completed.

### DTI MRI

For DTI MRI, a series of sagittal spin-echo multislice diffusion-weighted images were acquired. A spin-echo sequence was used to enhance the signal-to-noise ratio of each image. The MRI parameters were: TR = 730 ms; TE = 72 ms; matrix = 128 × 64; FOV = 60 × 60 mm; slice thickness = 1.5 mm; scan time = 180 min; b-value = 0.601 s/mm^2^; duration of gradient pulse/diffusion time (δ/Δ) = 10/15 ms; directions = 6; number of signal average (NEX) = 32.

### MR image analysis

ADC, MD, and FA maps were obtained using FSL imaging software (FSL.5.0.6)^30^. To accurately measure MRI signal intensity, ADC, MD, and FA in four portions (CO, OS, IS, and IM) were determined using Image J software and its Restore Selection function as described^9^. Use of this function resulted in successful selection of the exact same portions of the kidney anatomically referenced in standard T_2_-weighted images for measuring MRI signal intensity, ADC, MD, and FA.

### Western blotting analysis

Western blotting was performed as described^31^. After samples were boiled for 10 min, the supernatants were separated in 4–20% gradient or 7% sodium dodecyl sulfate-polyacrylamide gels and transferred to polyvinylidene fluoride membranes (GE Healthcare Japan, Tokyo, Japan). The membranes were washed three times in TBST (20 mM Tris-HCl pH 8.0, 150 mM NaCl, 0.05% Tween 20) for 5 min each, blocked for 30 min in Blocking-One (Nacalai Tesque, Kyoto, Japan), and washed three times with TBST for 5 min each. The membranes were then incubated for 1 h at room temperature with antibodies against α-smooth muscle actin (α-SMA) (Abcam, Tokyo, Japan; 1:500) and collagen I (Abcam; 1:500) in Can-get-signal (Toyobo, Osaka, Japan), washed with three times with TBST for 10 min each, incubated with peroxidase-conjugated anti-mouse IgG or anti-rabbit IgG (Dako, Tokyo, Japan; 1:5,000) in Can-get-signal for 1 h, and washed three times with TBST for 10 min each. Signals were developed using ECL plus reagent (GE Healthcare Japan) and detected using an LAS imager (GE Healthcare Japan).

### Quantitative PCR (qPCR) analysis

One-half of each harvested kidney was homogenized in a Polytron tissue homogenizer (PT 1300D; Kinematica AG, Luzern, Switzerland). The mRNA was extracted with TRIzol solution (Life Technologies, Carlsbad, CA, USA) and reverse-transcribed to cDNA using a PrimeScript RT-PCR Kit (Takara, Shiga, Japan). Real-time PCR was performed with SYBR Premix Ex Taq (Takara) and a Thermal Cycler Dice Real Time System Single (TP850; Takara), according to the manufacturer’s instructions, using primers for rat transforming growth factor (TGF)-β, α-SMA, connective tissue growth factor (CTGF), and tissue inhibitor of metalloproteinase (TIMP)-1, as described^32^. Real-time PCR data were processed and evaluated by an unpaired *t*-test using Multiplate RQ software (Takara).

### Pathological staining and immunohistochemistry

For renal histological analyses, one-half of each kidney was fixed in 4% paraformaldehyde in phosphate-buffered saline and embedded in paraffin. Tissue slices of 4 μm were stained with Masson trichrome^33^ and Sirius Red^34^.

### RF analyses

Conventional methods for measuring RF use Masson trichrome staining of randomly obtained magnified fields, followed by image analysis with Image J software. However, because of possible biases in selecting fields, a Biorevo BZ-9000 microscope (Keyence, Osaka, Japan) and BZ-X analyzer software (Keyence) were used. After automated capture of 40x magnified images over the entire tissue sample, a single high-resolution image of the whole kidney was created seamlessly (Supplementary Fig. S6a,b). Each whole kidney image was dissected into four portions (CO, OS, IS, and IM), and the renal fibrosis in each portion was quantified by Micro Cell Count software (Keyence) using common parameters (Supplementary Fig. S6c–f). Sirius Red staining was used instead of Masson trichrome staining, because the former is more specific for collagen types I and III^35^. DAB based immunohistochemistry (collagen type I or α-SMA) was not used, because the software cannot be applied to the heterogeneous background staining observed in DAB based immunohistochemistry.

### Statistical analysis

Unpaired and nonparametric *t*-tests (Mann–Whitney tests) were used to compare two different kidney tissues, while one-way analysis of variance and Dunnett’s multiple comparison test were used to compare three or more samples or kidney tissues. All statistical analyses were performed using GraphPad Prism 6 software (GraphPad Software Inc., San Diego, CA, USA).

## Electronic supplementary material

Supplementary Information

## References

[1] Currie G, McKay G, Delles C (2014). Biomarkers in diabetic nephropathy: Present and future. World J Diabetes.

[2] Bohle A, Mackensen-Haen S, von Gise H (1987). Significance of tubulointerstitial changes in the renal cortex for the excretory function and concentration ability of the kidney: a morphometric contribution. Am J Nephrol.

[3] Risdon RA, Sloper JC, De Wardener HE (1968). Relationship between renal function and histological changes found in renal-biopsy specimens from patients with persistent glomerular nephritis. Lancet.

[4] Wild S, Roglic G, Green A, Sicree R, King H (2004). Global prevalence of diabetes: estimates for the year 2000 and projections for 2030. Diabetes Care.

[5] Park CW (2014). Diabetic kidney disease: from epidemiology to clinical perspectives. Diabetes Metab J.

[6] Gonzalez Suarez ML, Thomas DB, Barisoni L, Fornoni A (2013). Diabetic nephropathy: Is it time yet for routine kidney biopsy?. World J Diabetes.

[7] Artunc F, Rossi C, Boss A (2011). MRI to assess renal structure and function. Curr Opin Nephrol Hypertens.

[8] De Keyzer, F. T. & H. C. *Extra-Cranial Applications of Diffusion-Weighted MRI*. 32–45 (Cambridge University Press, 2011).

[9] Kaimori JY (2013). Non-invasive magnetic resonance imaging in rats for prediction of the fate of grafted kidneys from cardiac death donors. PLoS One.

[10] Togao O (2010). Assessment of renal fibrosis with diffusion-weighted MR imaging: study with murine model of unilateral ureteral obstruction. Radiology.

[11] Inoue T (2011). Noninvasive evaluation of kidney hypoxia and fibrosis using magnetic resonance imaging. J Am Soc Nephrol.

[12] Boor, P. *et al*. Diffusion-weighted MRI does not reflect kidney fibrosis in a rat model of fibrosis. *J Magn Reson Imaging*, doi:10.1002/jmri.24853 (2015). 10.1002/jmri.24853 25630829

[13] Hunsche S, Moseley ME, Stoeter P, Hedehus M (2001). Diffusion-tensor MR imaging at 1.5 and 3.0 T: initial observations. Radiology.

[14] Pfefferbaum A (2000). Age-related decline in brain white matter anisotropy measured with spatially corrected echo-planar diffusion tensor imaging. Magn Reson Med.

[15] Liu Z (2015). Chronic kidney disease: pathological and functional assessment with diffusion tensor imaging at 3T MR. Eur Radiol.

[16] Feng Q, Ma Z, Wu J, Fang W (2015). DTI for the assessment of disease stage in patients with glomerulonephritis–correlation with renal histology. Eur Radiol.

[17] Lu L (2011). Use of diffusion tensor MRI to identify early changes in diabetic nephropathy. Am J Nephrol.

[18] Fan, W. J. *et al*. Assessment of renal allograft function early after transplantation with isotropic resolution diffusion tensor imaging. *Eur Radiol*, doi:10.1007/s00330-015-3841-x (2015). 10.1007/s00330-015-3841-x 26017738

[19] Hueper K (2012). Magnetic resonance diffusion tensor imaging for evaluation of histopathological changes in a rat model of diabetic nephropathy. Invest Radiol.

[20] Kaimori JY (2017). Diffusion Tensor Imaging MRI With Spin-Echo Sequence and Long-Duration Measurement for Evaluation of Renal Fibrosis in a Rat Fibrosis Model. Transplant Proc.

[21] Watanabe D (2009). Renoprotective effects of an angiotensin II receptor blocker in experimental model rats with hypertension and metabolic disorders. Hypertens Res.

[22] Ohmura T (2012). Renoprotective effects of telmisartan on renal injury in obese Zucker rats. Acta Diabetol.

[23] Field AS, Wu YC, Alexander AL (2005). Principal diffusion direction in peritumoral fiber tracts: Color map patterns and directional statistics. Ann N Y Acad Sci.

[24] Yan YY (2017). Intravoxel incoherent motion and diffusion tensor imaging of early renal fibrosis induced in a murine model of streptozotocin induced diabetes. Magn Reson Imaging.

[25] Zhang JL (2010). Variability of renal apparent diffusion coefficients: limitations of the monoexponential model for diffusion quantification. Radiology.

[26] Hori M (2008). Mean diffusivity, fractional anisotropy maps, and three-dimensional white-matter tractography by diffusion tensor imaging. Comparison between single-shot fast spin-echo and single-shot echo-planar sequences at 1.5 Tesla. Eur Radiol.

[27] Cheung JS (2010). Diffusion tensor imaging of liver fibrosis in an experimental model. J Magn Reson Imaging.

[28] Saksena S (2008). Cerebral diffusion tensor imaging and *in vivo* proton magnetic resonance spectroscopy in patients with fulminant hepatic failure. J Gastroenterol Hepatol.

[29] Hayashi T (2013). Diffusion analysis with triexponential function in liver cirrhosis. J Magn Reson Imaging.

[30] Smith SM (2004). Advances in functional and structural MR image analysis and implementation as FSL. Neuroimage.

[31] Hatanaka M (2016). Azilsartan Improves Salt Sensitivity by Modulating the Proximal Tubular Na+ -H+ Exchanger-3 in Mice. PLoS One.

[32] Moers C, van Rijt G, Ploeg RJ, Leuvenink HG (2012). The effect of normothermic recirculation before cold preservation on post-transplant injury of ischemically damaged donor kidneys. Transpl Int.

[33] Puchtler H, Waldrop FS, Valentine LS (1973). Polarization microscopic studies of connective tissue stained with picro-sirius red FBA. Beitr Pathol.

[34] Junqueira LC, Bignolas G, Brentani RR (1979). Picrosirius staining plus polarization microscopy, a specific method for collagen detection in tissue sections. Histochem J.

[35] Street, J. M. *et al*. Automated quantification of renal fibrosis with Sirius Red and polarization contrast microscopy. *Physiol Rep* **2**, doi:10.14814/phy2.12088 (2014). 10.14814/phy2.12088 PMC4187565 25052492

