# Supplementary material for: Visualization of kidney fibrosis in diabetic nephropathy by long diffusion tensor imaging MRI with spin-echo sequence
Source: Sci Rep. 2017 Jul 18;7:5731. doi: 10.1038/s41598-017-06111-4 (PMC5515876; doi:10.1038/s41598-017-06111-4)
Supplement: Supplementary file 1 — Supplementary Information [file 41598_2017_6111_MOESM1_ESM.pdf]

Supplementary Information of:

Visualization of kidney fibrosis in diabetic nephropathy by long diffusion tensor imaging MRI with spin echo sequence

Jun-ya Kaimori[1,2*], Yoshitaka Isaka[2], Masaki Hatanaka[2], Satoko Yamamoto[2], Naotsugu Ichimaru[1], Akihiko Fujikawa[3], Hiroshi Shibata[3], Akira Fujimori[3], Sosuke Miyoshi[3], Takashi Yokawa[4], Kagayaki Kuroda[5], Toshiki Moriyama[6], Hiromi Rakugi[2], Shiro Takahara[1]

[1] Department of Advanced Technology for Transplantation, Osaka University Graduate School of Medicine, Suita, Osaka, Japan

[2] Department of Geriatric Medicine and Nephrology, Osaka University Graduate School of Medicine, Suita, Osaka, Japan

[3] Drug Discovery Research, Astellas Pharma Inc., Tsukuba, Ibaraki, Japan

[4] BioView Inc., Chiyoda-ku, Tokyo, Japan

[5] Department of Human and Information Science Tokai University School of Information Science and Technology, Hiratsuka, Kanagawa, Japan

[6] Osaka University Health Care Center, Suita, Osaka, Japan

* Corresponding to:

Jun-Ya Kaimori M.D., PhD; E-mail: kaimori@att.med.osaka-u.ac.jp

a

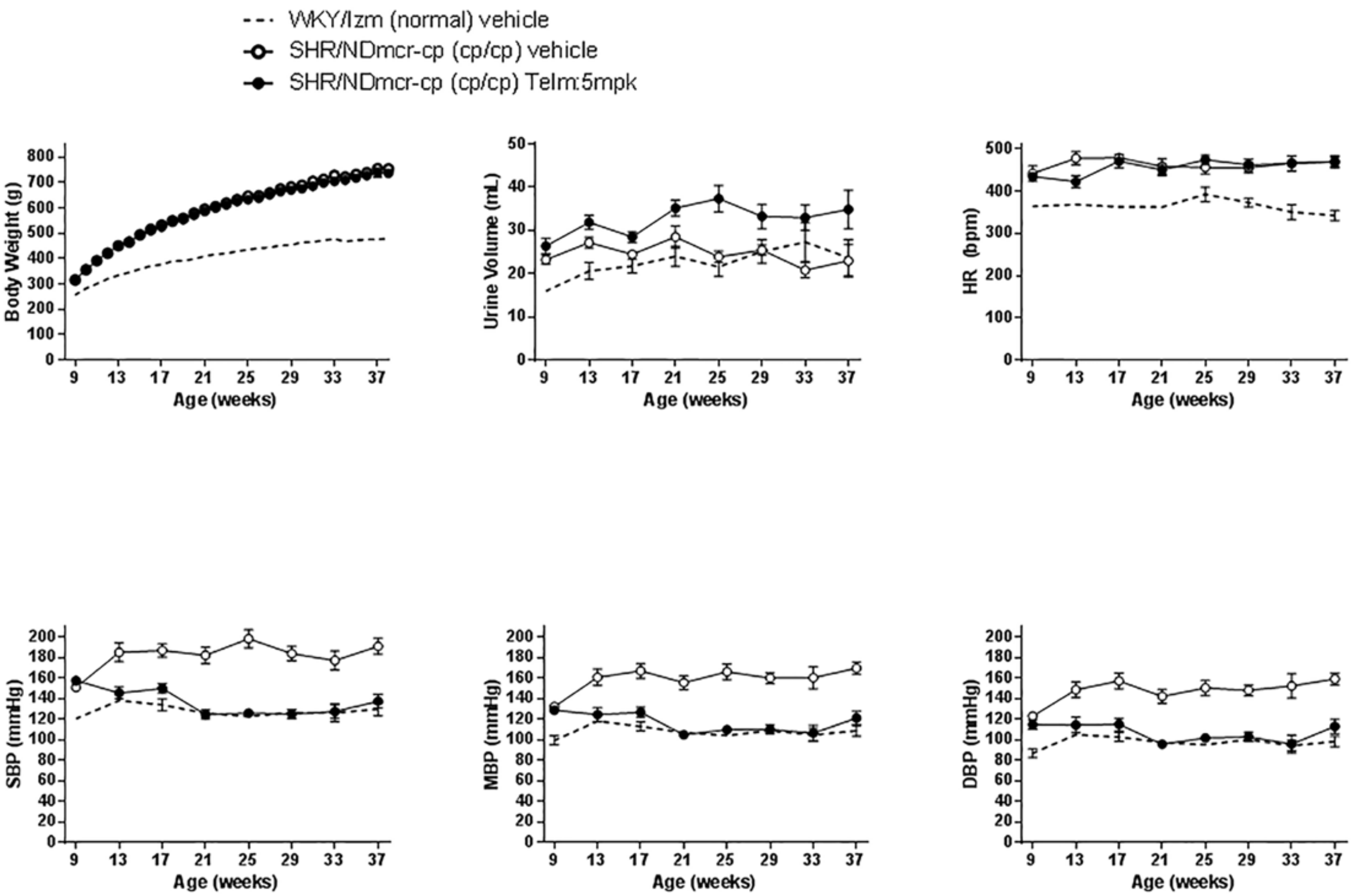

b

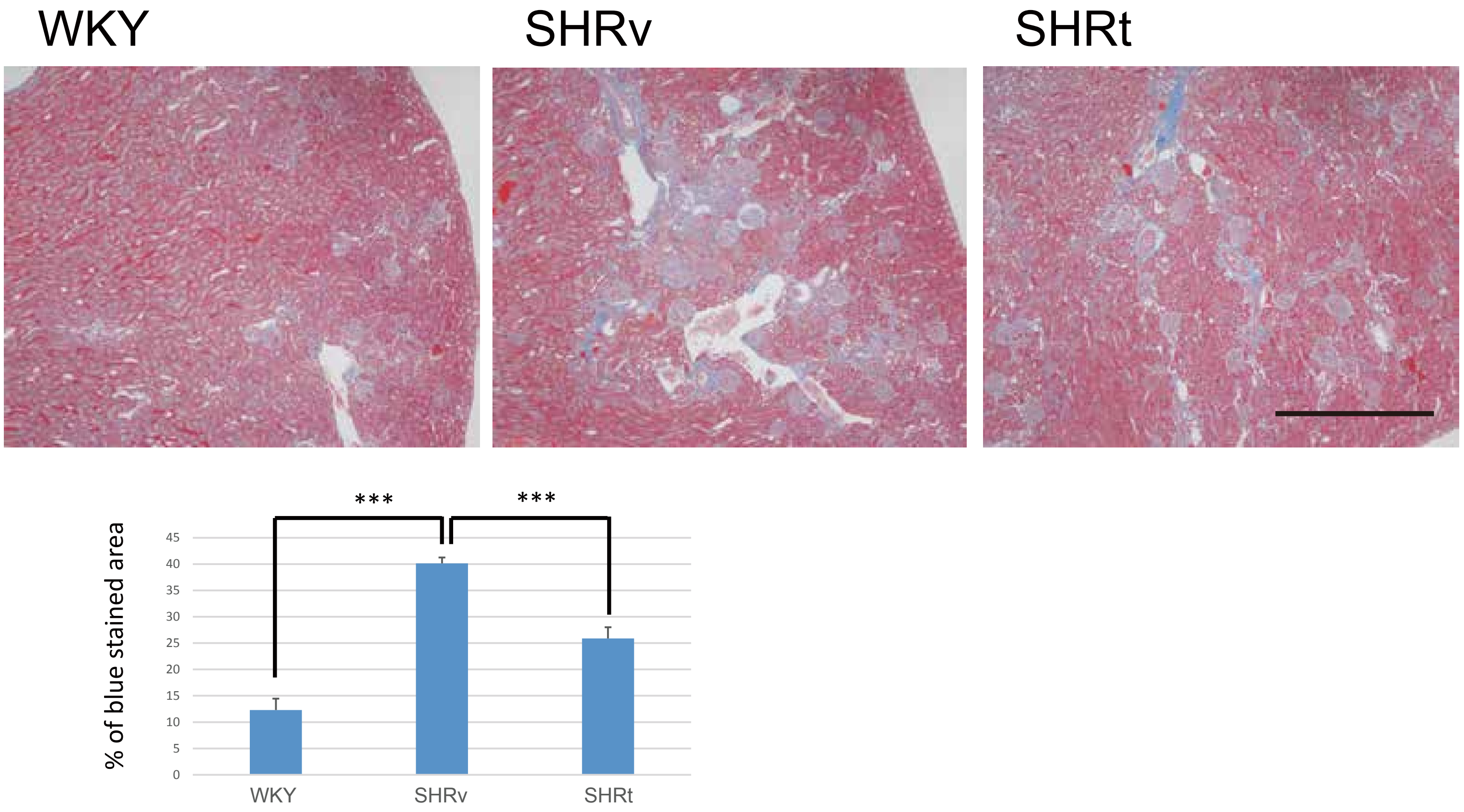

Supplementary Figure S1. Characterization of SHR/ND rats in a pilot study.
(a) Characterization of physiological properties in SHR/ND rats: body weight, urine volume, heart rate (HR), systolic blood pressure (SBP), mean blood pressure (MBP), and diastolic blood pressure (DBP) in WKY rats, SHR/ND rats treated with vehicle, and SHR/ND rats treated with telmisartan. The data are shown as mean ± SD.
(b) (upper) Masson trichrome staining of kidney tissues from WKY rats, SHR/ND rats treated with vehicle (SHRv), and SHR/ND rats treated with telmisartan (SHRt). Scale bar, 500 µm. (lower) Quantification of fibrotic areas in the kidney tissues of the rats. The data are shown as mean ± SD.

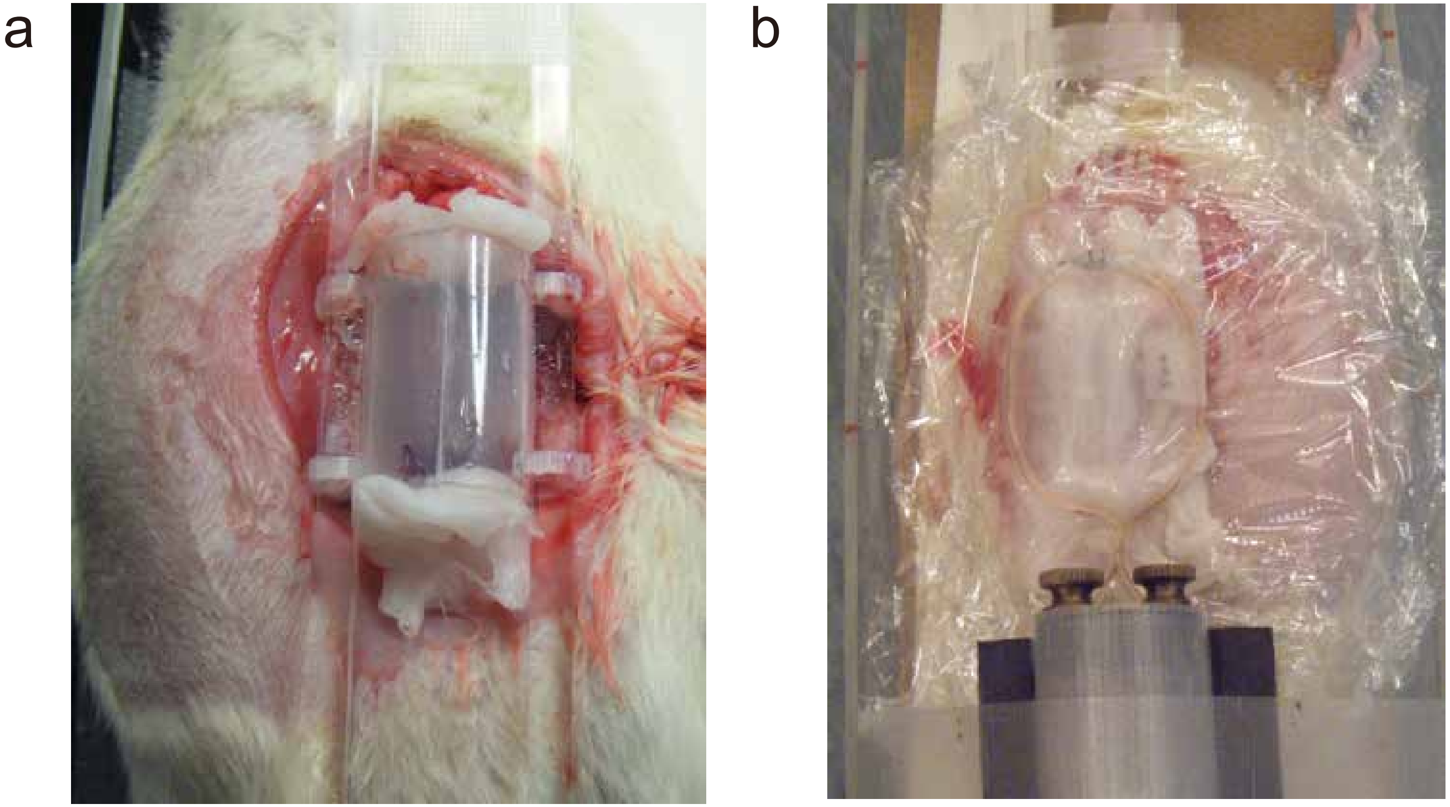

Supplementary Figure S2. Special attachment for the rat kidney for long MRI measurement.
(a) Kidney attachment used in WKY and SHR/ND rats. (b) MRI setting for both rats. The set was composed of an animal platform, a thin plastic film, and an in-house-made detection coil.

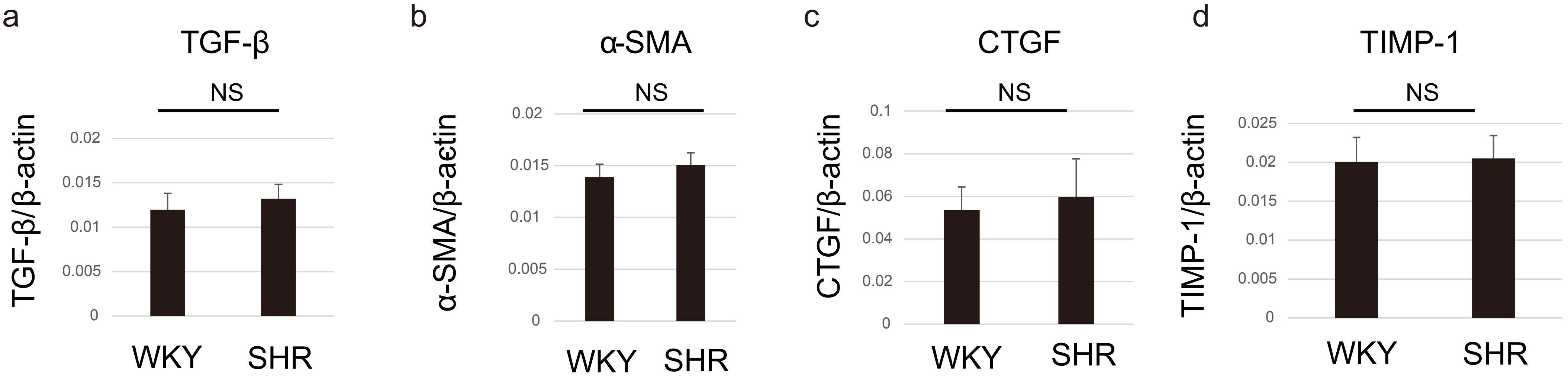

Supplementary Figure S3. qPCR assays of RF-related genes.
(a–d) Expression levels of TGF-β (a), α-SMA (b), CTGF (c), and TIMP-1 (d) mRNAs in WKY and SHR/ND rat kidneys. The data are shown as mean ± SD.

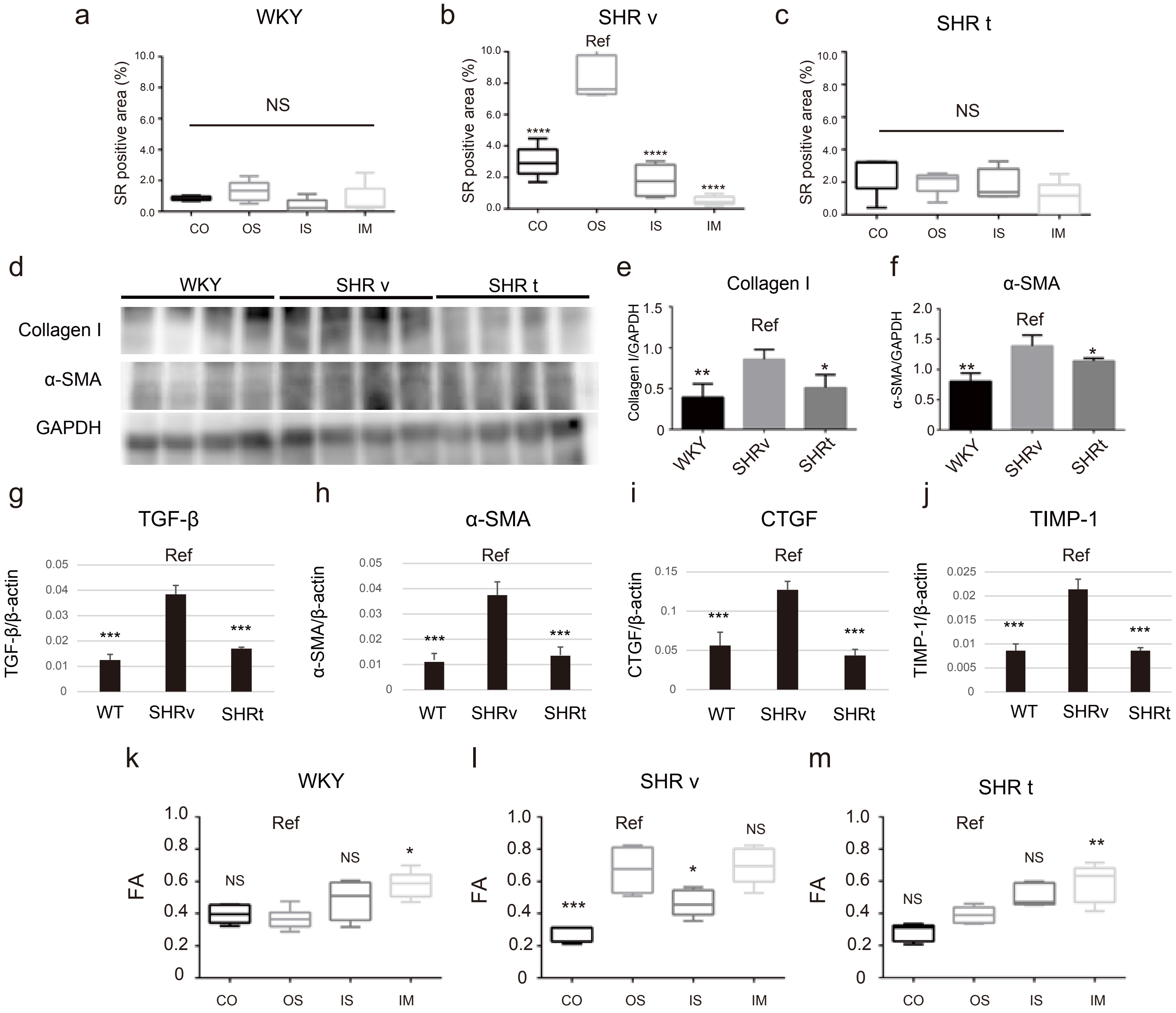

Supplementary Figure S4. Biochemical and pathological evaluation of RF.
(a–c) Quantification of Sirius Red (SR) staining of the CO, OS of outer medulla, IS of outer medulla, and IM of kidneys from WKY rats (a), SHR/ND rats treated with vehicle (SHRv) (b), and SHR/ND rats treated with telmisartan (SHRt) (c). ***:$p<0.001$, OS vs other portions. The data are shown as mean ± SD. (d) Western blotting for expression of collagen I and α-SMA in WKY (left panel), SHRv (middle panel), and SHRt (left panel) kidneys. The uncropped western blots are shown in Supplementary Fig. S7. (e,f) Quantification of expression of collagen I (e) and α-SMA (f) in the kidneys normalized by the expression of glyceraldehyde-3-phosphate dehydrogenase (GAPDH). (g–j) qPCR results for expression of TGF-β (g), α-SMA (h), CTGF (i), and TIMP-1 (j) mRNAs in WKY (left), SHRv (middle), and SHRt (left) kidneys. *:$p<0.05$,**:$p<0.01$,***:$p<0.001$, SHRv vs WKY or SHRt. FA values in different portions of WKY (k), SHRv (l), and SHRt (m) kidneys. ***:$p<0.001$, OS vs other portions. The data are shown as mean ± SD.

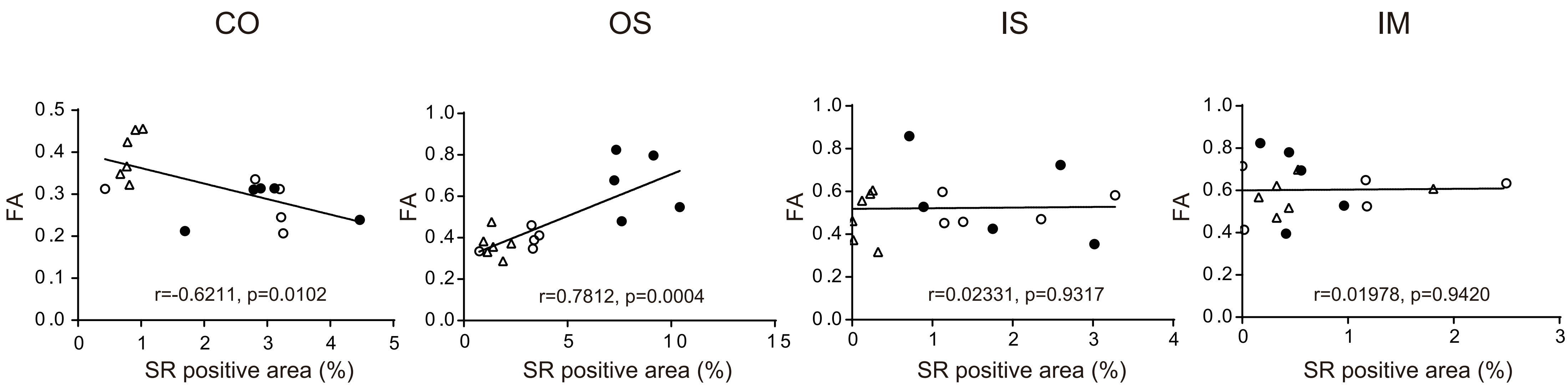

Supplementary Figure S5. Correlations of FA values with fibrotic areas measured by SR staining based on the data of Figure 4d and WKY/Ism rats. Open circles, closed circles and open triangles indicate the data from SHRt, SHRv and WKY/Ism rats respectively.

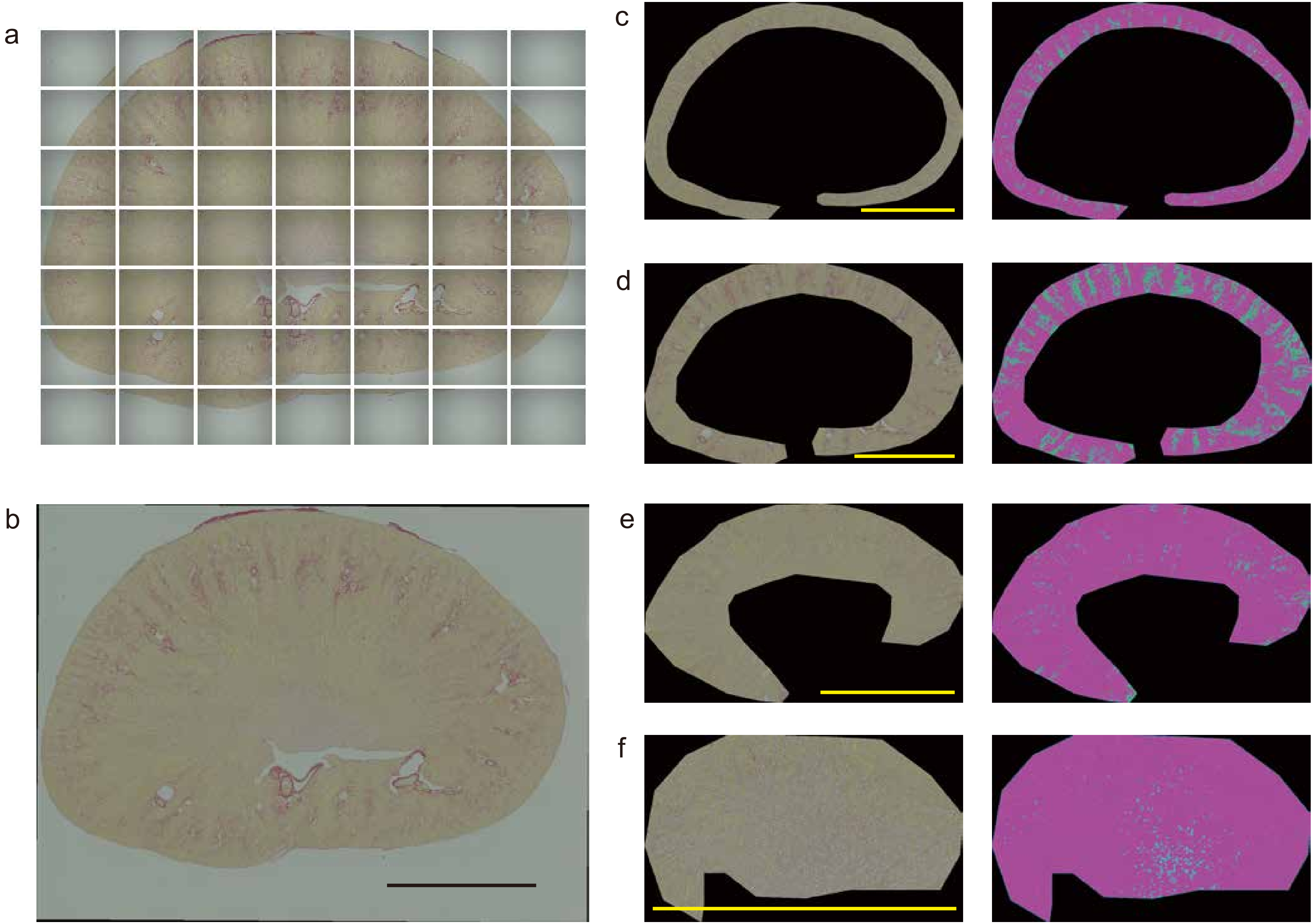

Supplementary Figure S6. Semi-automated RFs measurement. (a,b) Forty-two magnified Sirius red-stained images captured by an automated microscope (a) and a single image of the whole kidney created from these images (b). Scale bar, 5 mm. (c–f) The image of each whole kidney was divided into four kidney portions comprising the CO (c), OS (d), IS (e), and IM (f) (left panels), and RF was quantified in each portion using Micro Cell Count software (right panels).

Collagen I

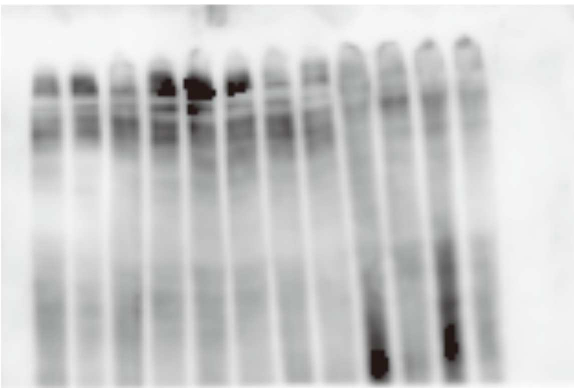

GAPDH

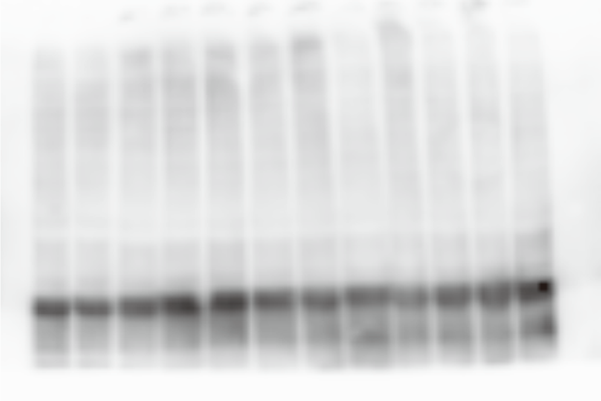

α-SMA

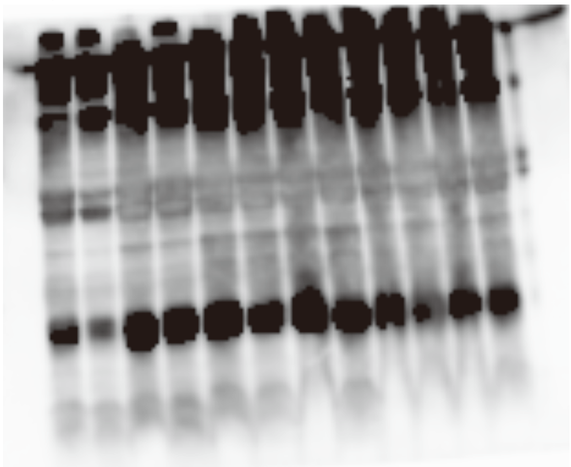

Supplementary Figure S7. Uncropped western blots of Supplementary Figure S4d.
